# New Approved Drugs Appearing in the Pharmaceutical Market in 2022 Featuring Fragments of Tailor-Made Amino Acids and Fluorine

**DOI:** 10.3390/molecules28093651

**Published:** 2023-04-22

**Authors:** Nana Wang, Haibo Mei, Gagan Dhawan, Wei Zhang, Jianlin Han, Vadim A. Soloshonok

**Affiliations:** 1 Jiangsu Co-Innovation Center of Efficient Processing and Utilization of Forest Resources, College of Chemical Engineering, Nanjing Forestry University, Nanjing 210037, China; 2 School of Allied Medical Sciences, Delhi Skill and Entrepreneurship University, Dwarka, New Delhi 110075, India; 3 Department of Biomedical Science, Acharya Narendra Dev College, University of Delhi, Kalkaji, New Delhi 110019, India; 4 Delhi School of Skill Enhancement and Entrepreneurship Development, Institution of Eminence, University of Delhi, Delhi 110007, India; 5 Department of Chemistry, University of Massachusetts Boston, 100 Morrissey Boulevard, Boston, MA 02125, USA; 6 Department of Organic Chemistry I, Faculty of Chemistry, University of the Basque Country UPV/EHU, Paseo Manuel Lardizábal 3, 20018 San Sebastián, Spain; 7 IKERBASQUE, Basque Foundation for Science, Alameda Urquijo 36-5, 48011 Bilbao, Spain

**Keywords:** fluorine-containing compounds, blockbuster drugs, pharmaceuticals, drug design and development, tailor-made amino acids

## Abstract

The strategic fluorination of oxidatively vulnerable sites in bioactive compounds is a relatively recent, widely used approach allowing us to modulate the stability, bio-absorption, and overall efficiency of pharmaceutical drugs. On the other hand, natural and tailor-made amino acids are traditionally used as basic scaffolds for the development of bioactive molecules. The main goal of this review article is to emphasize these general trends featured in recently approved pharmaceutical drugs.

## 1. Introduction

**Figure 1:**
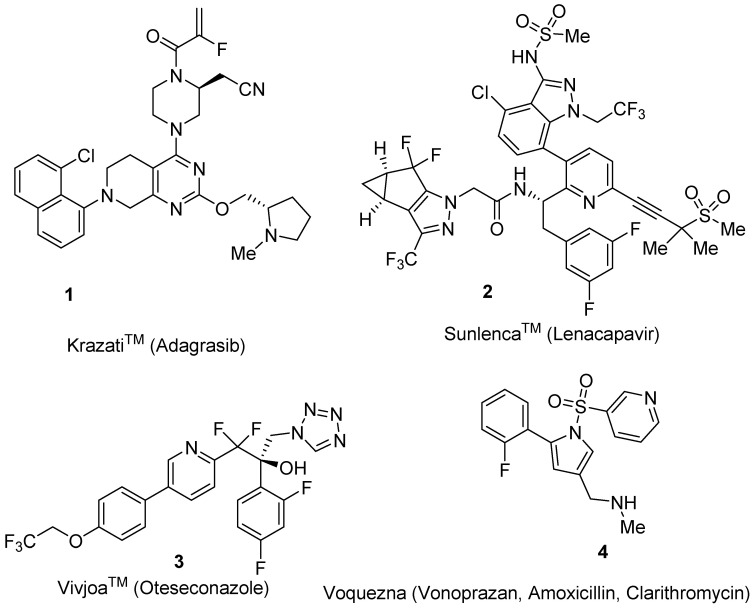
Fluorine-containing drugs approved by the FDA in 2022.

**Figure 2:**
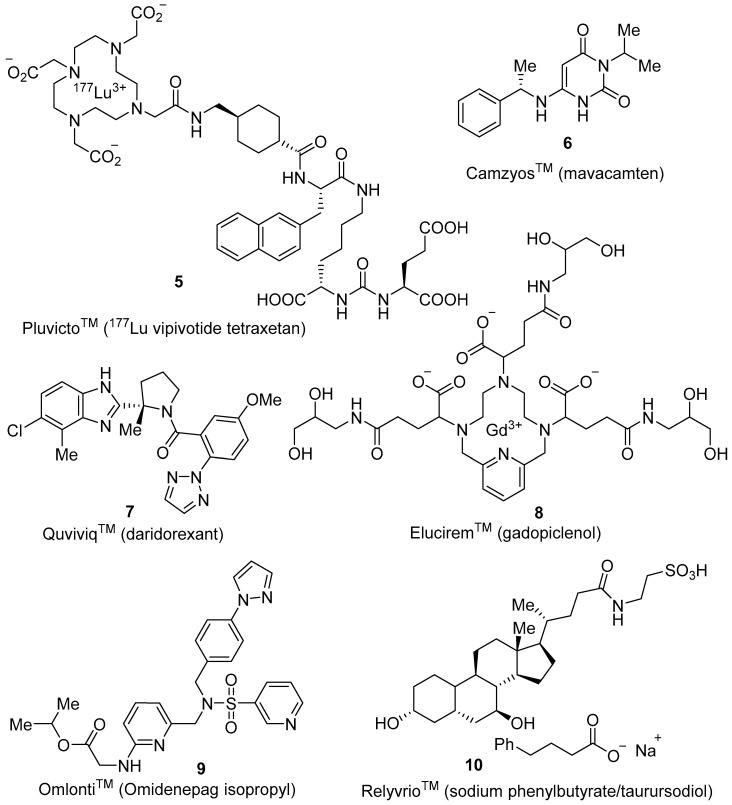
Tailor-made amino-acid-derived drugs.

Modern pharmaceutical drugs feature tremendous molecular variety in terms of size, shape, and chemical functionalities. Nevertheless, thorough structural analysis allows us to find two clear similarities: a framework derived from a parent amino acid (AA), and the presence of fluorine [1,2]. Being ubiquitous naturally occurring compounds, AAs have traditionally played an important role in areas of life sciences, such as the development of new pharmaceuticals, medicinal formulations, biosensors, and drug delivery systems [3,4,5,6,7,8,9,10]. Indeed, in the modern paradigms of medicinal chemistry and drug discovery, tailor-made AAs [11] are indispensable components increasingly found in newly marketed pharmaceutical products [12,13,14,15,16,17,18]. Thus, over 30% of small-molecule drugs contain residues of tailor-made AAs or amino-alcohols and di-amines derived from them [12,13,14,15,16,17,18]. In contrast to AAs, the building blocks of life, fluorine is essentially a xenobiotic element [19,20,21], with nearly zero footprint in biochemical evolution. Nevertheless, since the discovery of fludrocortisone in 1953 [22,23,24]—the first Food and Drug Administration (FDA)-approved fluorine-containing drug—the idea of introducing fluorine into biologically active compounds has attracted the close attention of the pharmaceutical industry. Nowadays, over 30% of marketed drugs contain at least one fluorine atom [25,26,27,28,29,30,31,32]. Quite naturally, chemistry practitioners constantly pay very special attention to the records relevant to new pharmaceutical drugs, particular aspects of their structural design, and therapeutic areas. Considering the current role of tailor-made AAs and fluorine in the development of modern drugs, one may agree that the discussion of compounds featuring these two traits might be of keen interest to the appropriate scientific community. The goal of this review article is to profile 10 (Figure 1 and Figure 2) out of 22 FDA-approved small-molecule drugs, all new tailor-made AA-derived/fluorine-containing drugs introduced to the market in 2022. For each compound, the general mode of biological activity and synthetic routes are presented. 

## 2. Fluorine-Containing Drugs

### 2.1. Adagrasib (Krazati^TM^)

Adagrasib (**1**, MRTX849; Krazati), a potent and selective KRAS inhibitor of the RAS GTPase family, was developed by Mirati Therapeutics as an anticancer compound to treat non-small cell lung cancer (NSCLC). The molecule specifically targets cysteine 12 residue, the most common KRAS mutation [33], and the compound inhibits the downstream signaling pathway and demonstrates anti-tumor activity. In February 2022, the FDA accepted a new drug application filing for adagrasib (**1**) for the treatment of patients with previously treated KRASG12C-positive NSCLC. Further, in December 2022, the FDA granted accelerated approval to adagrasib for the treatment of KRASG12C-mutated NSCLC patients who have received at least one prior systemic therapy [34,35,36,37,38,39,40,41].

**Figure 3:**
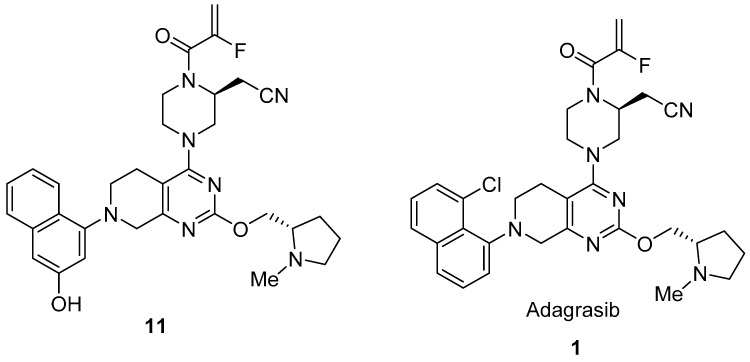
Structural comparison of **11** and adagrasib (**1**).

A series of analogs with tetrahydropyrimidine moieties have been reported in the literature to act as irreversible covalent inhibitors of KRASG12C [35,36]. Compound **11** was reported as an irreversible covalent inhibitor binding cysteine12 in the binding pocket of KRAS. The pharmacokinetic limitations of **11** led to the development of adagrasib (**1**) (Figure 3). The rational drug discovery approach to identify the title compound **1** began with the observation that removal of the hydroxyl group from **11** resulted in a fivefold improvement in oral bioavailability. Further, optimization to increase potency was performed after visualizing the crystal structure of the dehydroxy analog complexed to KRASG12C wherein a bound water molecule was complexed to Gly10 and Thr58, and the displacement of this water could lead to an increase in potency. Further optimization led to the 8-chloro analog with an IC_50_ value of 1 nM. The title compound **1**, having a 2-fluoroacrylamide group, provides increased half-life across species due to a decrease in GSH metabolism while maintaining potency (IC_50_ = 5–14 nM).

**Scheme 1:**
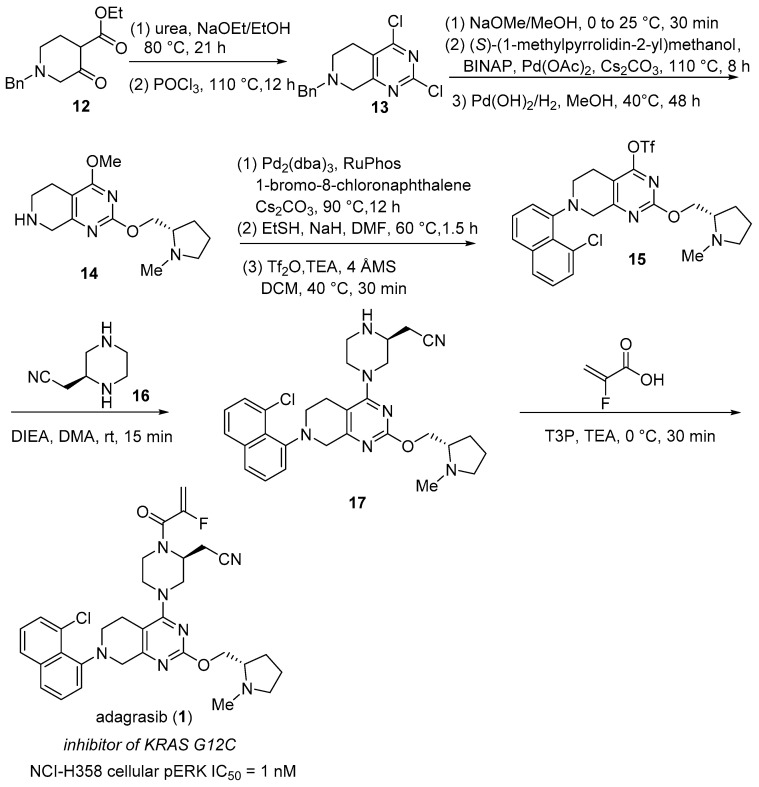
Synthesis of adagrasib (**1**).

The synthesis of adagrasib (**1**) is shown in Scheme 1 [35,36]. The first step is the condensation of the starting material **12** and urea to provide the bicyclic dione core, which is followed by chlorination with POCl_3_ to provide **13**. The Buchwald coupling reaction is employed, wherein the C2 prolinol side chain is attached, followed by benzyl hydrogenolysis to give compound **14**. The intermediate **14** is converted to 8-chloronaphthyl substituted intermediate **15**, which then undergoes displacement of trifluoromethanesulfonate (OTf) by (S)-2-(piperazin-2-yl)acetonitrile (**16**) to afford the intermediate **17**. Finally, amidation of compound **17** with propylphosphonic anhydride (T3P) as the coupling reagent affords adagrasib (**1**).

### 2.2. Lenacapavir (Sunlenca^TM^)

Lenacapavir (**2**) is a first-in-class human immunodeficiency virus (HIV) drug known as a capsid inhibitor that can be used in combination with other antiretroviral drugs as a twice-yearly treatment strategy developed by Gilead Sciences. It was approved by the FDA in December 2022 for HIV-1 inhibition to treat adults with multi-drug-resistant HIV infection, and it functions by preventing HIV from multiplying, thereby reducing virion levels in the body [42,43,44,45,46,47,48,49,50,51]. It contains a difluorobenzyl ring that occupies the same phenylalanine-glycine binding pocket as polyadenylation specificity factor subunit 6 (CPSF6) and nucleoporin 153 (Nup153), which establishes extensive hydrophobic and hydrogen bonding interactions, thereby interrupting the capsid protein interactions with Nup153 and CPSF6 [52,53,54,55,56].

**Scheme 2:**
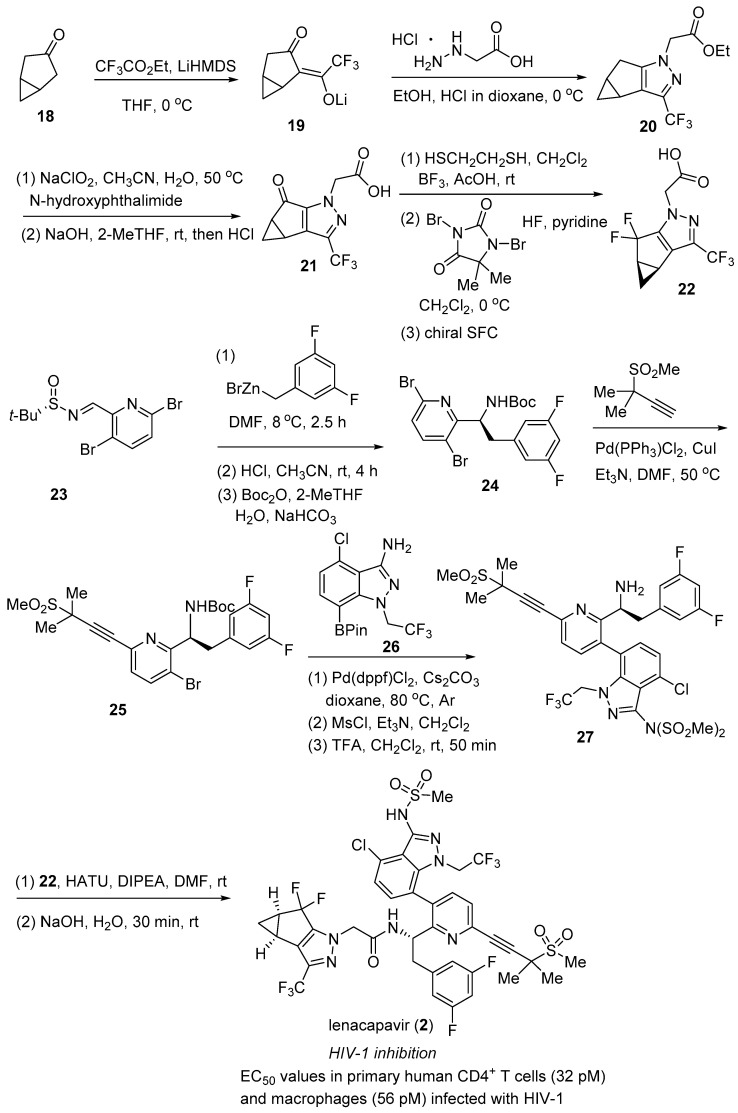
Synthesis of lenacapavir (**2**).

The synthesis of lenacapavir (**2**) is shown in Scheme 2 [57]. The starting bicyclo[3.1.0]hexan-3-one (**18**) is treated with lithium hexamethyldisilazide (LIHMDS) and reacted with ethyl 2,2,2-trifluoroacetate to give the enolate **19**, which undergoes a cyclization reaction with hydrazinoacetic acid in HCl to afford pyrazole intermediate **20**. The intermediate **20** is oxidized by *N*-hydroxyphthalimide and NaClO_2_ to yield compound **21**, which is subjected to a deoxyfluorination reaction to afford **22** after chiral SFC separation. On the other hand, imine **23** undergoes an addition reaction to yield chiral intermediate **24**, which is subjected to a Sonogashira coupling reaction with 3-methyl-3-(methylsulfonyl)but-1-yne, affording the alkyne intermediate **25**. Then, Suzuki coupling of **25** with borate **26** gives alkyne **27**, which couples with the intermediate **22** in the presence of 2-(7-azabenzotriazol-1-yl)-N,N,N′,N′-tetramethyluronium hexafluorophosphate (HATU) and diisopropylethylamine (DIPEA) to afford the targeted lenacapavir (**2**).

### 2.3. Oteseconazole (Vivjoa™)

**Figure 4:**
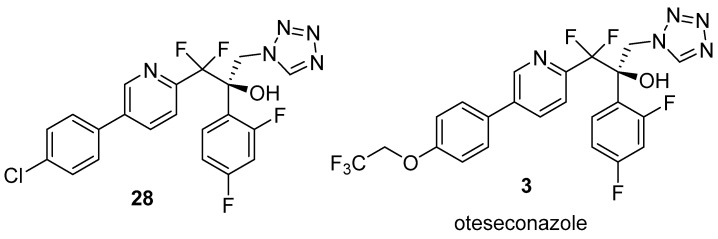
Structures of oteseconazole (**3**) and its analog.

Oteseconazole (**3**) is an effective oral antifungal agent developed by Mycovia Pharmaceuticals [58]. It can inhibit cytochrome P450 (CYP51), thus affecting the formation and integrity of fungal cell membranes. The binding strength of oteseconazole to CYP51 is generally similar to that of other azole antifungal agents, including fluconazole, which inhibits CYP51 activity in a manner consistent with tight binding inhibition. However, compared with other azole antibacterial agents, oteseconazole does not show inhibitory activity of human CYP51 [59,60]. Study results have confirmed the effectiveness of oteseconazole in the treatment of the initial episode of vulvovaginal candidiasis (VVC) and strengthened its effectiveness and safety in the treatment of recurrent vulvovaginal candidiasis (RVVC) compared with the current standard-care drug, fluconazole, for VVC [61]. Oteseconazole is a chiral compound that contains a difluoromethyl-pyridine unit, a tetrazole heterocyclic moiety, and a difluorophenyl group at the carbinol center (Figure 4). Structure–activity relationship (SAR) studies by Viamet Pharmaceuticals Inc. disclosed that the substitution of trifluoroethyl ether by a chloro group led to decreased inhibitory activity against Trichophyton rubrum (T. rubrum) (T. rubrum MIC values of <0.001 and 0.004 for compounds **3** and **28**, respectively) [62]. On April 26, 2022, the FDA approved the oral antifungal drug Vivjoa (oteseconazole) to reduce the incidence rate of RVVC in women [58].

**Scheme 3:**
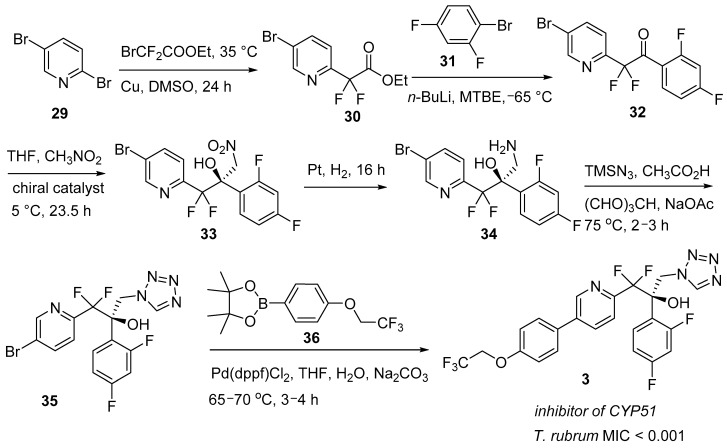
Synthesis of oteseconazole (**3**).

The preparation of oteseconazole (**3**) is shown in Scheme 3 using 2,5-dibromopyridine (**29**) as the starting material [63]. Cu-promoted coupling reaction of 2,5-dibromopyridine with bromodifluoroacetate affords ethyl 2-(5-bromopyridin-2-yl)-2,2-difluoroacetate (**30**), which undergoes a substitution reaction with a lithium reagent in situ generated from 1-bromo-2,4-difluorobenzene (**31**), providing the ketone intermediate **32**. Then, the asymmetric Henry reaction of ketone **32** gives the nitro compound **33**, which is subjected to a Pt-catalyzed reduction reaction. Cyclization reaction of the amine intermediate **34** with trimethylsilylazide (TMSN_3_) affords the intermediate **35**. Finally, Suzuki coupling reaction of compound **35** with 4,4,5,5-tetramethyl-2-(4-(2,2,2-trifluoroethoxy)phenyl)-1,3,2-dioxaborolane (**36**) gives the targeted oteseconazole (**3**).

### 2.4. Vonoprazan/Amoxicillin/Clarithromycin (Voquezna^TM^)

Vonoprazan (**4**) was developed by Takeda Corporation of Japan and approved for the treatment of gastroesophageal reflux disease (GERD) in Japan on 16 December 2014 [64]. Vonoprazan (**4**) contains a fluorophenyl unit and a pyridin-3-ylsulfonyl pyrrole ring. It is a potassium-competitive acid blocker to inhibit the acid secretion rate of gastric parietal cells [65]. Because vonoprazan (**4**) has a long half-life and longer action time, it is considered an effective long-term proton pump inhibitor (PPI) [66]. The earliest randomized double-blind phase III experiment showed that the eradication rate of Helicobacter pylori (Hp) in the population with a vonoprazan protocol was 92.6%, while the eradication rate of Hp in the population with a lansoprazole protocol was 75.9% [67,68,69]. On 3 May 2022, vonoprazan (**4**) combined with amoxicillin and clarithromycin was approved by the FDA with the trade name Voquezna^TM^ for the treatment of adult Hp infection. These approvals were supported by the results from phase 3 of the phalcon-EE double-blind trial.

**Scheme 4:**
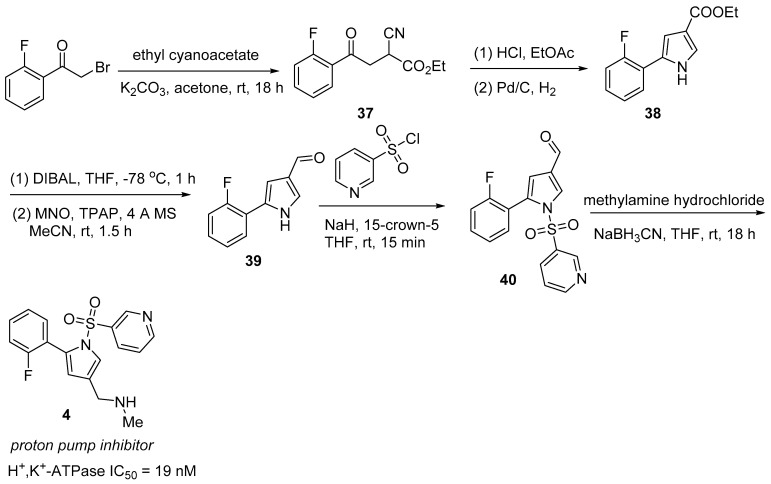
Synthesis of vonoprazan (**4**).

The synthesis of vonoprazan (**4**) is shown in Scheme 4 [70], using the corresponding α-bromoacetophenone derivative as the starting material. The first step is the condensation reaction of 2-bromo-1-(2-fluorophenyl)ethan-1-one with ethyl cyanoacetate in the presence of potassium carbonate, affording the intermediate **37**. Then, a cyclization reaction of the intermediate **37** via treatment with hydrochloric acid results in 5-arylpyrrole-3-carboxylic acid ester **38**. Subsequently, the reduction of ester **38** by diisobutyl aluminum hydride (DIBAL) followed by oxidation in the presence of tetra-*n*-propylammonium perruthenate (TPAP) and *N*-methylmorpholine *N*-oxide (MNO) provides the aldehyde **39**. The obtained aldehyde intermediate **39** is sulfonylated by pyridine-3-sulfonyl chloride with NaH as a base to generate the intermediate **40**. Finally, a reductive amination reaction of intermediate **40** using methylamine hydrochloride gives vonoprazan (**4**).

## 3. AA-Derived Drugs

### 3.1. ^177^Lu Vipivotide Tetraxetan (Pluvicto^TM^)

^177^Lu vipivotide tetraxetan (**5**), also known as ^177^Lu PSMA-617, is a small molecule designed to bind with prostate-specific membrane antigen (PSMA) [71,72,73]. Pluvicto uses high-affinity targeting ligands to guide effective radiotherapy to prostate cancer cells. The specific target of this therapy comes from the “ligand” part of the therapeutic agent. The PSMA-targeted ligand in Pluvicto is chemically connected to a therapeutic radioactive atom called Lutetium-177 (^177^Lu), which releases high-energy β particles to accurately transmit cytotoxic radiation to the disease site [74]. Different from traditional external radiotherapy, Pluvicto is administered by systemic injection, which could directly target multiple PSMA-positive prostate cancer sites throughout the body, including bones and soft tissues. On March 23, 2022, FDA approved Pluvicto for the treatment of adult patients with PSMA-positive metastatic castration-resistant prostate cancer (mCRPC) who have received androgen receptor pathway inhibition and taxane-based chemotherapy. These regulatory decisions were supported by the key phase III VISION study results, in which the death risk of PSMA-positive mCRPC patients receiving Pluvicto plus standard treatment was statistically significantly reduced [75].

**Scheme 5:**
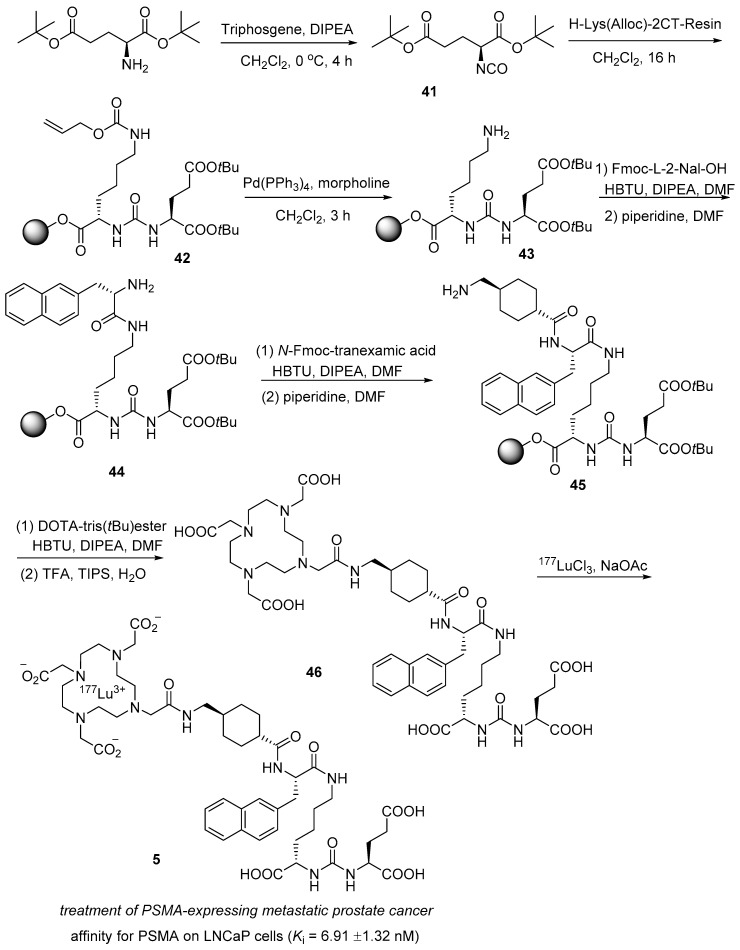
Synthesis of ^177^Lu vipivotide tetraxetan (**5**).

^177^Lu vipivotide tetraxetan (**5**) contains several amino acid units, including glutamate, lysine, 3-(2-naphthyl)-L-alanine, and trans-4-(aminomethyl)cyclohexanecarboxylic acid. Thus, the preparation of ^177^Lu vipivotide tetraxetan (**5**) can proceed via traditional solid-phase peptide synthesis (Scheme 5) [76,77]. First, isocyanate **41** is obtained via the reaction of bis(*tert*-butyl) L-glutamate hydrochloride with triphosgene in the presence of DIPEA at 0 °C. Then, isocyanate **41** is reacted with resin-immobilized (2-chloro-tritylresin, Merck) ε-allyloxycarbonyl-protected lysine, generating the intermediate **42**. Removal of the allyloxycarbonyl-protecting group via treatment with Pd(PPh_3_)_4_ and morpholine in CH_2_Cl_2_ affords the compound **43**. Subsequently, L-2-Nal-OH and tranexamic acid are introduced via a condensation reaction in the presence of *O*-(benzotriazol-1-yl)-*N*,*N*,*N*′,*N*′-tetramethyluronium hexafluorophosphate (HBTU) and DIPEA, and the peptide intermediate **45** is obtained. Then, the reaction of intermediate **45** with DOTA-tris(*t*Bu)ester, followed by removal of *tert*-butyl and cleavage of the resin via treatment with trifluoroacetic acid (TFA), triisopropylsilane (TIPS), and water, provides PSMA-617 (**46**). Finally, the reaction of compound **46** with LuCl_3_ gives the target product **5**.

### 3.2. Mavacamten (Camzyos^TM^)

Mavacamten (**6**) is an oral selective allosteric inhibitor of cardiac myosin adenosine triphosphate (ATP) enzyme; it was the world’s first innovative therapeutic drug directly targeting the pathophysiological mechanism of hypertrophic cardiomyopathy (HCM) [78,79,80]. It can reduce the contraction force of sarcomeres and reversibly inhibit the coupling reaction between myosin and actin by inhibiting MYH7 mutation, which leads to an increase in myosin ATPase activity. Mavacamten (**6**) can reduce the sensitivity of the myocardium to Ca^2+^, which may be due to it delaying the formation of the cross bridge and accelerating the separation of the cross bridge, so that the myocardial contractility can return to normal. At the same time, it can also promote the whole myosin group to change into an energy-saving super-relaxation state, and improve diastolic function and energy metabolism [81,82]. On April 28, 2022, mavacamten (**6**) was approved by the FDA with the name Camzyos^TM^ to treat adults with symptomatic New York Heart Association (NYHA) Class II-III obstructive hypertrophic cardiomyopathy to improve functional ability and symptoms. Camzyos is the first and only FDA-approved allosteric and reversible inhibitor of cardiac myosin, targeting the potential pathophysiology of obstructive HCM [83].

**Scheme 6:**
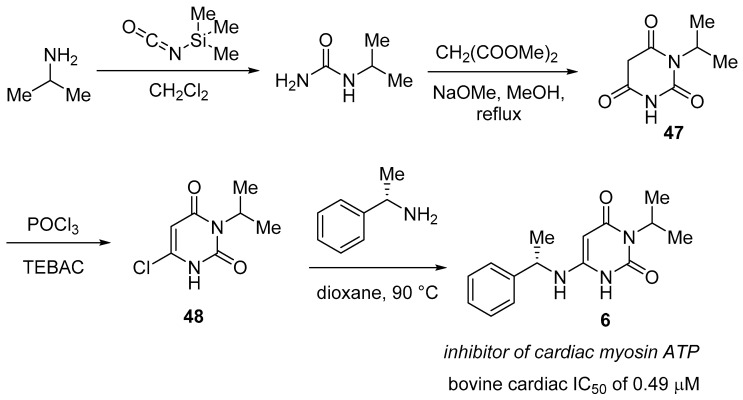
Synthesis of mavacamten (**6**).

The synthetic method of mavacamten (**6**) is described in Scheme 6 with isopropylamine as the starting material [84]. Isopropylamine is reacted with trimethylsilyl isocyanate to give 1-isopropylurea, which is then refluxed with dimethyl malonate and sodium methoxide at 65 °C, resulting in 1-isopropyl barbituric acid (**47**). Chlorination of compound **47** by POCl_3_ in the presence of triethylbenzyl ammonium chloride (TEBAC) at 50 °C affords the pale yellow solid 6-chloro-3-isopropylpyridine-2,4 (1*H*,3*H*)-dione (**48**). Finally, the desired mavacamten (**6**) is obtained by stirring compound **48** with methylaniline at 90 °C for 24 h.

### 3.3. Daridorexant (Quviviq^TM^)

Daridorexant (**7**) is a dual orexin receptor (DOR) antagonist, developed by the Swiss biotechnology company Idorsia, that is used to treat adult patients with insomnia. Daridorexant plays a hypnotic role by blocking the binding of neuropeptides orexin A and orexin B with receptors OX_1_R and OX_2_R [85,86]. The results of a phase III clinical trial showed that daridorexant significantly improved the total sleep time by comparison with placebo in the first and third months of treatment [87]. Daridorexant (**7**) received approval from the FDA on 7 January 2022 with the trade name Quviviq [88].

**Scheme 7:**
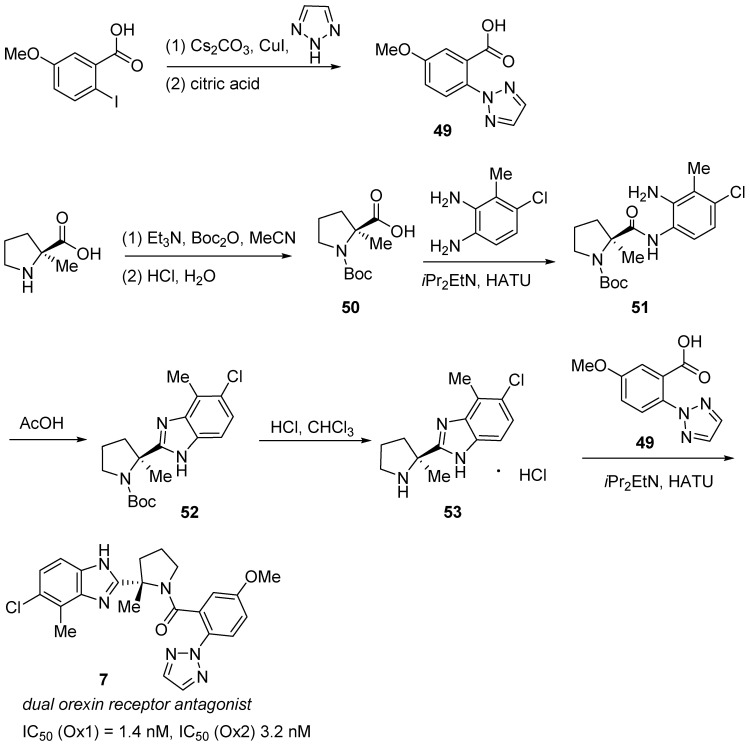
Synthesis of daridorexant (**7**).

The synthesis of daridorexant is described in Scheme 7, using (*S*)-2-methylpyrrolidine-2-carboxylic acid hydrochloride as the starting material [89]. First, one key triazole intermediate **49** is prepared via a Cu-catalyzed cross-coupling reaction of 2-iodo-5-methoxybenzoic acid and 2*H*-1,2,3-triazole. On the other hand, the amino group of (*S*)-2-methylpyrrolidine-2-carboxylic acid is protected by *tert*-butyloxy carbonyl (Boc) in the presence of trimethylamine. The generated Boc-protected intermediate **50** undergoes a condensation reaction with 4-chloro-3-methylbenzene-1,2-diamine in the presence of *i*Pr_2_EtN and HATU, providing the intermediate **51**. Then, intramolecular cyclization of intermediate **51** generates imidazole intermediate **52**, which is subjected to a reaction with hydrochloric acid to give the free amine **53**. The target compound daridorexant (**7**) is finally obtained by a condensation reaction of intermediates **49** and **53** in the presence of DIPEA and HATU.

### 3.4. Gadopiclenol (Elucirem^TM^)

Gadopiclenol (**8**, Elucirem, Villepinte) is a paramagnetic, extracellular, nonspecific macrocyclic gadolinium-based contrast agent (GBCA) developed by Guerbet’s Research and Development team. Gadopiclenol (**8**) is a large-membered cyclic compound, featuring a 3,6,9-triaza-1(2,6)-pyridinacyclodecaphane unit and glutaric moiety (Figure 2). Gadopiclenol develops a magnetic moment when placed in a magnetic field. The magnetic moment alters the relaxation rates of water protons in its vicinity in the body, leading to an increase in the signal intensity of tissues and enhancing the magnetic resonance imaging (MRI) quality for tissue differentiation in disease diagnosis. The FDA approved gadopiclenol (**8**) in September 2022 primarily based on data obtained from phase III studies showing that gadopiclenol could improve image quality in brain and body MRI at half the conventional gadolinium dose [90].

The precursor for the preparation of perfusion computerized tomography with acetazolamide challenge (PCTA) derivatives (including gadopiclenol) is the Gd complex of PCTA known as Gd(PCTA-tris-glutaric acid). Gadopiclenol (**8**) is obtained by amidation of the above compound with isoserinol [91,92]. Gd(PCTA-tris-qlutaric acid) has three stereocenters on the glutaric moieties, leading to eight possible stereoisomers. However, the chemical structure of gadopiclenol contains a total of six stereocenters, and the exact composition of the isomeric mixture obtained, isomer separation, and isomer characterization were not provided or disclosed.

**Scheme 8:**
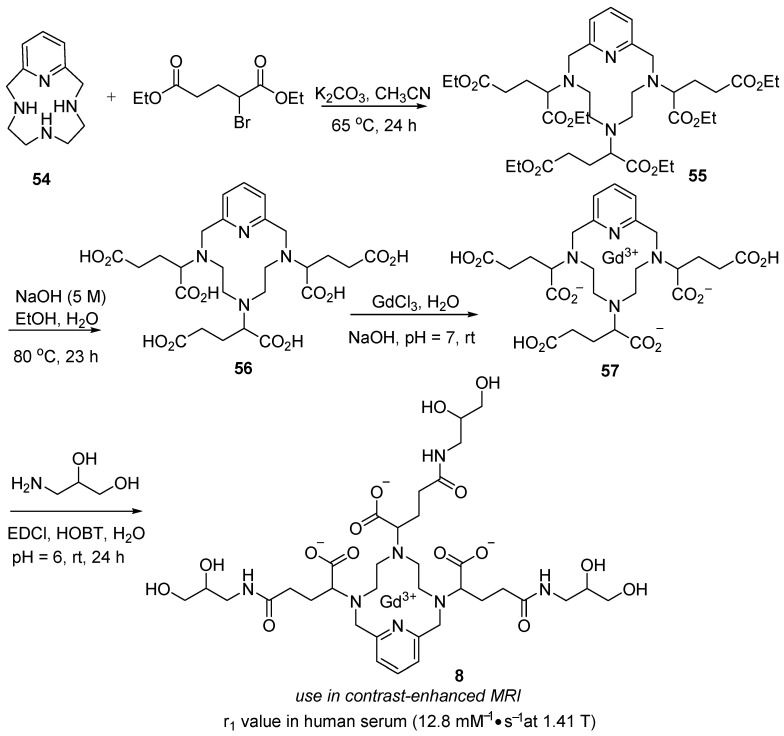
Synthesis of gadopiclenol (**8**).

The synthesis of Gd(PCTA-tris-glutaric acid) **57** and gadopiclenol (**8**) is shown in Scheme 8 [92]. Pyclen (3,6,9-triaza-1(2,6)-pyridinacyclodecaphane) (**54**) is alkylated with 3 equiv of diethyl 2-bromopentanedioate to give hexaester **55**, which is saponified to generate the corresponding hexacarboxylic acid derivative **56**. The polyacid **56** is complexed with 1 equiv of GdCl_3_ for Gd(PCTA-tris-glutaric acid) **57**, and the complex **57** is used in a peptidic coupling reaction with 3 equiv of 3-aminopropane-isoserinol to yield the desired hydrophilic and stable macrocyclic chelate gadopiclenol (**8**).

### 3.5. Omidenepag Isopropyl (Omlonti^TM^)

**Figure 5:**
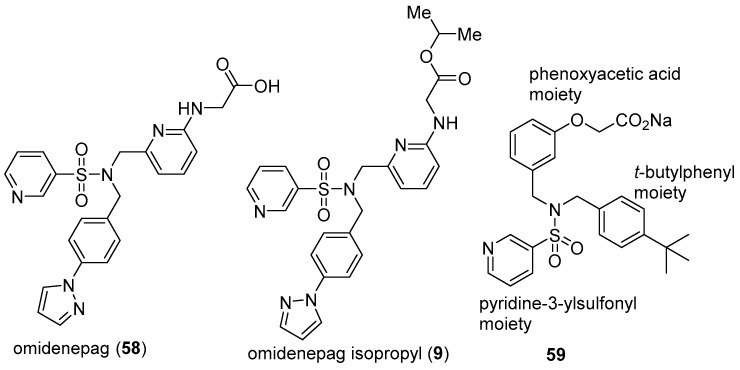
Structures of omidenepag (**58**), omidenepag isopropyl (**9**), and compound **59**.

Omidenepag (**58**) is a prostaglandin EP2 receptor agonist [93,94,95,96,97] developed by Santen pharmaceuticals (Figure 5). The compound **59** was reported as a selective EP2 receptor agonist containing a sulfonamide group, phenoxyacetic acid moiety, pyridin-3-ylsulfonyl moiety, and *tert*-butylphenyl moiety. It was identified as the lead compound in quantitative structure–activity relationship (QSAR) efforts [98,99,100]. Further, it was reported that the phenoxyacetic acid moiety is a very critical substructure for its biological activity [101,102]. Compound **58** with a (pyridin-2-ylamino)acetic acid substructure substituted at the 6-position of the pyridyl group showed higher h-EP2 receptor agonist activity than compound **59**. Omidenepag (**58**) containing a pyrazol-1-yl group displayed the most potent h-EP2 receptor agonist activity compared to compound **59** [103,104,105,106,107,108,109]. Taking into consideration the above facts, SAR efforts were made by modifying the phenoxyacetic acid, pyridin-3-ylsulfonyl, and *tert*-butylphenyl moieties of compound **59**. The results led to the development of omidenepag isopropyl (**9**), demonstrating potent and selective activity toward the human EP2 receptor (h-EP2) with an EC_50_ value of 1.1 nM. Omidenepag isopropyl (**9**) was approved by the FDA in September 2022 with the indication of reducing elevated intraocular pressure in patients with open-angle glaucoma, and it could thus be used as an ocular hypotensive agent for intraocular pressure (IOP).

**Scheme 9:**
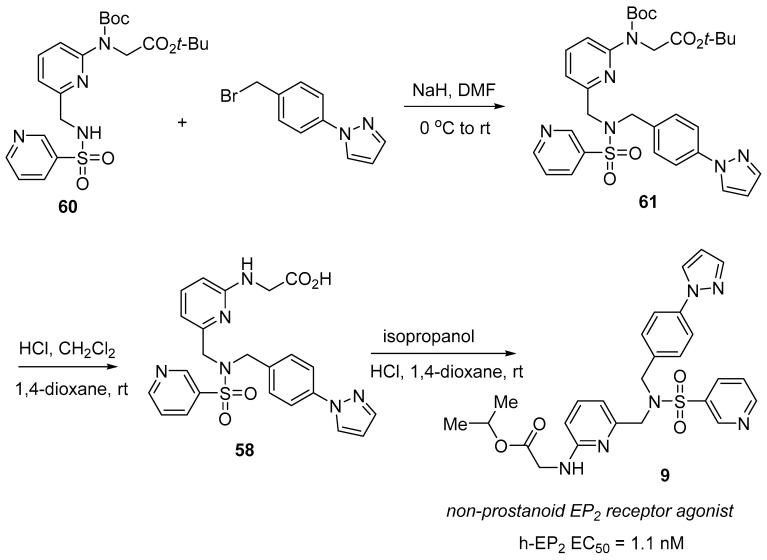
Synthesis of omidenepag isopropyl (**9**).

The synthetic route for omidenepag isopropyl (**9**) is shown in Scheme 9 [109]. Compound **60** is reacted with 1-(4-(bromomethyl)phenyl)-1*H*-pyrazole in the presence of NaH in DMF under basic conditions, affording compound **61**, which is then converted into omidenepag (**58**) via deprotection of the Boc and the *t*-Bu groups under acidic conditions. Further conversion of omidenepag (**58**) in the presence of hydrochloric acid gives the desired omidenepag isopropyl (**9**) as a white solid.

### 3.6. Phenylbutyrate–Taurursodiol (Relyvrio^TM^)

**Figure 6:**
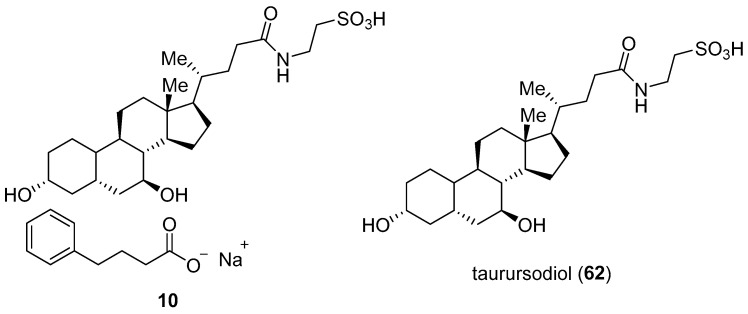
Structures of phenylbutyrate–taurursodiol **10** and taurursodiol (**62**).

Phenylbutyrate–taurursodiol (**10**, sodium phenylbutyrate/taurursodiol) is a fixed-dose combination oral treatment developed by Amylyx Pharmaceuticals for slowing disease progression in amyotrophic lateral sclerosis (ALS) patients [110,111,112,113,114,115,116,117,118]. Taurursodiol (**62**), also known as tauroursodeoxycholic acid, is the bile acid taurine conjugate and a more hydrophilic form of ursodeoxycholic acid, produced naturally in the body (Figure 6). Taurursodiol is responsible for improving mitochondrial energy production and anti-apoptotic effects [117,118]. Sodium phenylbutyrate is a salt of 4-phenylbutyric acid (4-PBA) [113] that is used to treat urea cycle disorders [114]; it acts as a chemical chaperone, preventing protein aggregation [115,116]. The combination of phenylbutyrate–taurursodiol was approved for medical use in Canada as Albrioza in June 2022 and in the USA as Relyvrio in September 2022 [119].

**Scheme 10:**
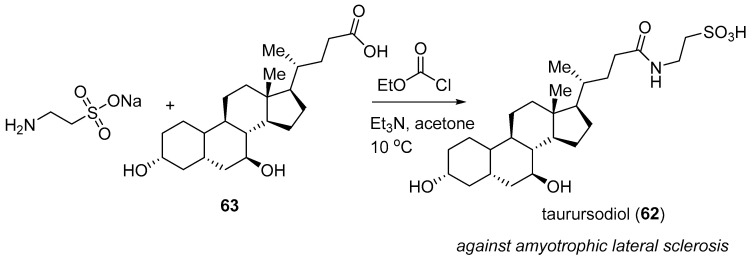
Synthesis of taurursodiol (**62**).

Sodium phenylbutyrate is prepared by reacting phenylbutyric acid with a sodium base [120]. Tauroursodeoxycholic acid **62** is prepared by selective precipitation of the impurities present in the suspension obtained from the reaction of an aqueous solution of sodium taurinate with an acetonic solution of a mixed anhydride of ursodeoxycholic acid **63** with an alkyl chloroformate (Scheme 10) [121].

## 4. Conclusions

From the standpoint of chemical structure, AAs represent an ideal platform for the rational design of modern pharmaceuticals. Thus, the presence of basic (amine) and acidic (carboxyl) functional groups, in combination with stereogenic carbon and practically unrestricted structural/functional space of the side chains, offers an extraordinary background for the design of a three-dimensional structural framework to achieve the desired biological functionality. Accordingly, one can expect that tailor-made AAs will continue to serve as indispensable building blocks in modern medicinal chemistry and drug design. As a result of the current and future importance of tailor-made AAs, there is clearly a fast-growing need in the availability of various structural types of AAs. Thus, the interest in new approaches for the asymmetric synthesis of tailor-made AAs is currently at an all-time high [122,123]. Some breakthrough developments have been made in the area of dynamic kinetic resolution of unprotected AAs [124,125], which can be efficiently used for large-scale synthesis and can compete with biocatalytic approaches in terms of affordability and low-cost structure. Nevertheless, the application of AAs has some inherent problematic issues. Some of them are the racemization of the stereogenic carbon, proteolytic and microsomal metabolism, clearance rates, and membrane permeability of AA-derived drugs. Fortunately, these issues can be ameliorated by the rational substitution of fluorine for hydrogen and/or the incorporation of fluorine-containing groups. The steric, electronic, and physical properties of the fluorinated groups [126,127,128,129] can be rationally applied to enhance configurational stability, reduce proteolytic and microsomal degradation, slow down clearance rates, and enhance membrane permeability [130,131,132], allowing us to quite successfully address the intrinsic stumbling blocks associated with the application of AAs.

## Data Availability

Not applicable.
